# Biofilm Filtrates of *Pseudomonas aeruginosa* Strains Isolated from Cystic Fibrosis Patients Inhibit Preformed *Aspergillus fumigatus* Biofilms via Apoptosis

**DOI:** 10.1371/journal.pone.0150155

**Published:** 2016-03-01

**Authors:** Fazal Shirazi, Jose A. G. Ferreira, David A. Stevens, Karl V. Clemons, Dimitrios P. Kontoyiannis

**Affiliations:** 1 Department of Infectious Diseases, Infection Control and Employee Health, The University of Texas M D Anderson Cancer Center, Houston, TX, 77030, United States of America; 2 Div. of Infectious Diseases and Geographic Medicine, Stanford University School of Medicine, Stanford, California, 94305, United States of America; 3 California Institute for Medical Research, San Jose, California, 95128, United States of America; University of Malaya, MALAYSIA

## Abstract

*Pseudomonas aeruginosa* (*Pa*) and *Aspergillus fumigatus* (*Af*) colonize cystic fibrosis (CF) patient airways. *Pa* culture filtrates inhibit *Af* biofilms, and *Pa* non-CF, mucoid (Muc-CF) and nonmucoid CF (NMuc-CF) isolates form an ascending inhibitory hierarchy. We hypothesized this activity is mediated through apoptosis induction. One *Af* and three *Pa* (non-CF, Muc-CF, NMuc-CF) reference isolates were studied. *Af* biofilm was formed in 96 well plates for 16 h ± *Pa* biofilm filtrates. After 24 h, apoptosis was characterized by viability dye DiBAc, reactive oxygen species (ROS) generation, mitochondrial membrane depolarization, DNA fragmentation and metacaspase activity. Muc-CF and NMuc-CF filtrates inhibited and damaged *Af* biofilm (p<0.0001). Intracellular ROS levels were elevated (p<0.001) in NMuc-CF-treated *Af* biofilms (3.7- fold) compared to treatment with filtrates from Muc-CF- (2.5- fold) or non-CF *Pa* (1.7- fold). Depolarization of mitochondrial potential was greater upon exposure to NMuc-CF (2.4-fold) compared to Muc-CF (1.8-fold) or non-CF (1.25-fold) (p<0.0001) filtrates. Exposure to filtrates resulted in more DNA fragmentation in *Af* biofilm, compared to control, mediated by metacaspase activation. In conclusion, filtrates from CF-Pa isolates were more inhibitory against *Af* biofilms than from non-CF. The apoptotic effect involves mitochondrial membrane damage associated with metacaspase activation.

## Introduction

Cystic fibrosis (CF) is an inherited disorder in which a mutation of the CF transmembrane conductance regulator gene (CFTR) leads to defective salt and water channels [1]. Despite significant progress in antimicrobial therapy and supportive care, chronic pulmonary infections and destructive inflammatory processes are still the major causes of morbidity and mortality of CF patients. CF patients have persistent colonization or infection with various opportunistic bacterial and fungal pathogens, of which *Pseudomonas aeruginosa* and *Aspergillus fumigatus* are the most prominent.

Both *A*. *fumigatus* and *P*. *aeruginosa* have been shown to form complex aggregates of microorganisms in vivo, growing as biofilms with an extracellular matrix. Biofilms form a physical barrier to host defenses, and their formation often results in resistance to antimicrobials [2–4]. Long-term colonization of CF patients with *P*. *aeruginosa* is associated with changes to the bacterium, including an increased mutation frequency with the temporal development of genotypic and phenotypic variants, such as a mucoid type, in the CF lungs [5]. Concurrent colonization with both *P*. *aeruginosa* and *A*. *fumigatus* in CF patients leads to further decline in pulmonary function compared to mono-colonization with either microbe [5]. Interestingly, in a murine pulmonary model, mice co-infected with *P*. *aeruginosa* and *A*. *fumigatus* had a higher survival rate than mice infected by *A*. *fumigatus* alone, suggesting that *P*. *aeruginosa* may secrete antifungal compounds [6]. Small molecules secreted by *Pseudomonas* can inhibit biofilm formation by *Aspergillus* [5]. We have previously reported, in a study with 26 isolates of *P*. *aeruginosa*, that non-mucoid CF isolates (NMuc-CF) were more inhibitory to formation of *Aspergillus* biofilms or to preformed biofilms, than mucoid isolates (Muc-CF), and that mucoid isolates were more inhibitory than non-CF isolates [7]. In addition, it was noted that *P*. *aeruginosa* biofilm culture filtrates were more inhibitory against *A*. *fumigatus* biofilm formation than were *P*. *aeruginosa* filtrates from planktonic cultures.

In this study, our goal was to assess whether the inhibitory effects of biofilm culture filtrates from 3 different *P*. *aeruginosa* variants (i.e., Muc-CF, NMuc-CF, and non-CF), obtained from CF and non-CF patients, on *A*. *fumigatus* biofilms are mediated through induction of apoptosis.

## Materials and Methods

### Isolates and growth conditions

Representative *P*. *aeruginosa* isolates, Muc-CF, NMuc-CF, and non-CF types, were selected from homogeneous phenotype groups, as studied previously [7]. *P*. *aeruginosa* stocks were maintained at -80°C in Microbank microbial storage vials (Pro-Lab Diagnostics, Richmond Hill, Ontario, Canada). *P*. *aeruginosa* stock culture was initially inoculated on Trypticase Soy Agar plates containing 5% sheep blood agar (TSA; BBL, Becton Dickinson) and incubated overnight at 37°C.

To obtain the *P*. *aeruginosa* biofilm culture filtrates, a suspension of 10^4^ cells/mL of *P*. *aeruginosa*, were prepared in fresh RPMI 1640 medium without serum and allowed to adhere to tissue culture flasks (BD Biosciences, San Diego) for 2 h at 37°C on a 100 rpm shaker incubator (attachment phase). The medium was then removed and the flasks were rinsed gently 3 times with sterile saline. After washing, 20 ml of fresh RPMI-1640 was added to the flask and adhered cells formed *P*. *aeruginosa* biofilms in 24 h at 37°C. The medium was removed, transferred to a 50 ml conical tube, and centrifuged for 30 min at 2,000 x *g* to remove any suspended cells or debris. The biofilm supernatant was gently removed, filter sterilized (0.22 μm filter) (Fisher, Pittsburgh, PA) and used immediately.

*A*. *fumigatus* (reference strain 10*Af*) [7, 8] from frozen stock cultures was inoculated on potato dextrose agar plates and incubated for 72 hours at 37°C. The *A*. *fumigatus* conidia were harvested by flooding the surface of the agar plates with saline containing 0.05% (v/v) Tween 80. The conidia suspension was recovered and dispensed into a 50-mL conical centrifuge tube and stored at 4°C. Before use, the suspension was centrifuged for 15 minutes at 3000 x *g* at 4°C. The pellet was washed twice by centrifugation with sterile phosphate-buffered saline (PBS) and finally suspended in RPMI-1640.

To initiate biofilm formation, 100 μl of a suspension of *A*. *fumigatus* conidia was placed into each well (10^5^ conidia/well) of flat-bottom 96-well plates. When the wells were seeded, the entire microtiter plate was sealed with para-film and incubated for 16 h at 37°C in a shaker incubator at 100 rpm to allow the fungi to form biofilm. After biofilm formation, the medium was carefully aspirated, minimizing contact with the biofilm. The wells were washed 3 times with PBS, and 150 μl of fresh RPMI-1640 was added to each well. The plate was further incubated for 24 h at 37°C in a shaker incubator at 100 rpm to allow the fungi to form mature biofilms.

### Effect of *P*. *aeruginosa* culture filtrates on preformed *A*. *fumigatus* biofilm

#### A) XTT assay

To measure the activity of *P*. *aeruginosa* biofilm culture filtrate against *A*. *fumigatus* biofilms, the *Aspergillus* biofilm was grown for 16 h and the preformed biofilm was challenged with *Pseudomonas* filtrates (1:1 vol./vol. of *Pseudomonas* filtrate and RPMI) obtained from the non- CF, Muc-CF or NMuc-CF strains respectively, and were further incubated for 24 h at 37°C. After incubation, plates were washed with PBS, and metabolic activity was assessed with the use of the 2,3-Bis(2-methoxy-4-nitro-5-sulfophenyl)-2H-tetrazolium-5-carboxanilide (XTT) colorimetric formazan reduction assay. Briefly, 100 μl of XTT (1 mg) /menadione (0.17 mg) solution was added to each well. Plates were covered in aluminum foil and incubated in the dark for 2 h at 37°C. After incubation, 80 μl of the resulting colored supernatant from each well was removed and transferred into the corresponding wells of a new microtiter plate. Absorbance was recorded at 490 nm [9].

#### B) Viability assay

After 16 hours of incubation at 37°C, preformed *A*. *fumigatus* biofilm was exposed to *P*. *aeruginosa* non-CF, Muc-CF, or NMuc-CF biofilm filtrates. After 24 h of incubation with *P*. *aeruginosa* biofilm filtrates, the *A*. *fumigatus* biofilms were stained with the viability dye bis-[1,3-dibutylbarbituric acid] trimethine oxonol (DiBAC; Molecular Probes), which enters depolarized cells, as described previously [10, 11].

#### C) Detection of intracellular reactive oxygen species (ROS) in Aspergillus biofilms

After 16 h of *A*. *fumigatus* biofilm formation, intracellular reactive oxygen species (ROS) levels were measured in *A*. *fumigatus* biofilm exposed to *P*. *aeruginosa* filtrates, as previously described [11–16]. *A*. *fumigatus* biofilms were scraped from the plate and spiked with dihydrorhodamine (DHR) 123 (5.0 μg/ml). After 2 h at room temperature, the cells were washed with PBS, centrifuged at 13 000 x *g* for 5 minutes, harvested, and observed with a Nikon Microphot SA fluorescence microscope (excitation, 488 nm; emission, 520 nm). For quantitative assays, fluorescence intensity values were recorded by using a POLARstar Galaxy microplate reader (excitation, 488 nm; emission, 520 nm; BMG LABTECH.

#### D) Mitochondrial membrane potential measurement in A. fumigatus biofilms

Mitochondrial membrane depolarization was assessed by staining with rhodamine 123, a fluorescent dye that is distributed in the mitochondrial matrix, as described previously [11–16]. Briefly, preformed *A*. *fumigatus* biofilm scraped from a 96-well plate pre-exposed to *P*. *aeruginosa* filtrates (non-CF, Muc-CF, or NMuc-CF) for 24 h at 37°C, was harvested via centrifugation, washed twice, and resuspended in PBS. Rhodamine 123 was added to the final concentration of 10 μM, and the cells were incubated for 30 min in the dark at room temperature. Fluorescence intensity was recorded as described above (excitation, 488 nm; emission, 520 nm).

#### E) Measurement of DNA damage in A. fumigatus biofilms

DNA fragmentation, a characteristic of apoptosis, was detected using a terminal deoxynucelotidyltransferase-mediated dUTP nick end-labeling (TUNEL) assay [11–16]. After incubation for 16 h in RPMI-1640 medium, *A*. *fumigatus* biofilm was exposed to *P*. *aeruginosa* filtrate (non-CF, Muc-CF, or NMuc-CF) for 24 h at 37°C, fixed with 3.7% formaldehyde for 30 min on ice, and digested with a lysing enzyme mixture (0.25 mg/ml of chitinase, 15 U of lyticase, and 20 mg/ml lysing enzyme; Sigma-Aldrich) for 3 h at 30°C. The enzyme-digested cells were subjected to the TUNEL assay, as described in detail elsewhere [11–17]. The cells were observed for fluorescence as described above, with excitation and emission wavelengths of 488 nm and 520 nm, respectively.

#### F) Detection of metacaspase activity in Aspergillus biofilms

Activation of metacaspases was detected with the CaspACE FITC-VAD-FMK In Situ Marker (Promega), which fluoresces green when it binds to active metacaspases [14]. After 16 h of biofilm formation in RPMI-1640 medium, *A*. *fumigatus* biofilm was exposed to *P*. *aeruginosa* biofilm culture filtrates (non-CF, Muc-CF, or NMuc-CF) for 24 h at 37°C. The cells were then harvested, washed in PBS, and suspended in 10 μM CaspACE FITC-VAD-FMK solution. After 2 hours of incubation at room temperature, cells were washed twice and suspended in PBS. Samples were mounted and viewed in a fluorescence microscope as described above (emission, 488 nm; excitation, 520 nm).

### Statistical analysis

For all assays, three independent experiments were performed three times on three different days in triplicate. Multiple treatment groups were compared using a Kruskal-Wallis test and post-hoc paired comparisons were done using Dunnett’s tests. Calculations were made with InStat (GraphPad Software). All results are expressed as means ± standard deviations. Two-tailed *P* values of less than 0.05 were considered statistically significant.

## Results

### *P*. *aeruginosa* culture filtrates depresses metabolism and damages membranes of *A*. *fumigatus* preformed biofilm

**Fig 1:**
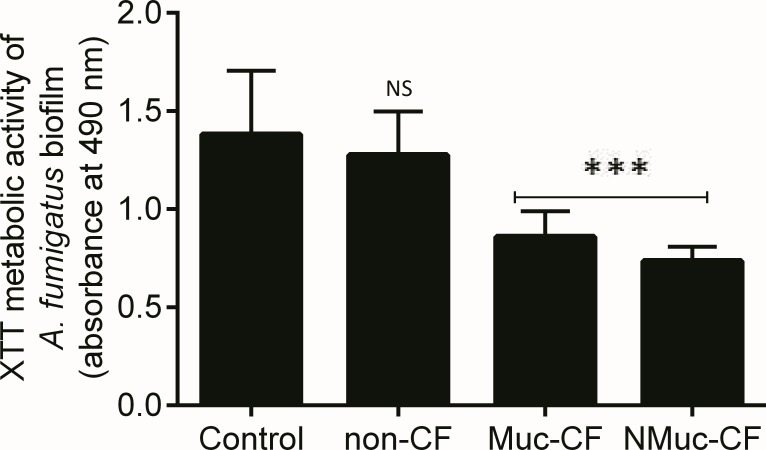
Inhibition of *A*. *fumigatus* biofilm formation of *P*. *aeruginosa* (*Pa*) biofilm culture filtrates: non-CF *Pa* isolate, mucoid CF *Pa* isolate (Muc-CF), and nonmucoid CF *Pa* isolate (NMuc-CF). Bars represent metabolic activity as measured by the XTT assay and by spectrophotometry at 490 nm. Assays were performed in triplicate and results are presented as the mean of three replicates. Error bars represent the standard deviation of the mean. ***p <0.0001 (compared with untreated controls).

Using the XTT reduction assay, we found that the biofilm culture filtrates of *P*. *aeruginosa* strains recovered from CF patients (Muc-CF and NMuc-CF) resulted in significant inhibition of growth of *A*. *fumigatus* preformed biofilm (Fig 1). The NMuc-CF- and Muc-CF-Pa biofilm filtrates- treated *Aspergillus* biofilms showed significantly greater inhibition than the untreated controls (p <0.0001) and the non-CF-Pa treated biofilms (p <0.0001). The non-CF *P*. *aeruginosa* culture filtrate was not inhibitory compared to control (p>0.05). *P*. *aeruginosa* culture filtrate from NMuc-CF was more inhibitory than that from Muc-CF (p>0.05).

**Fig 2:**
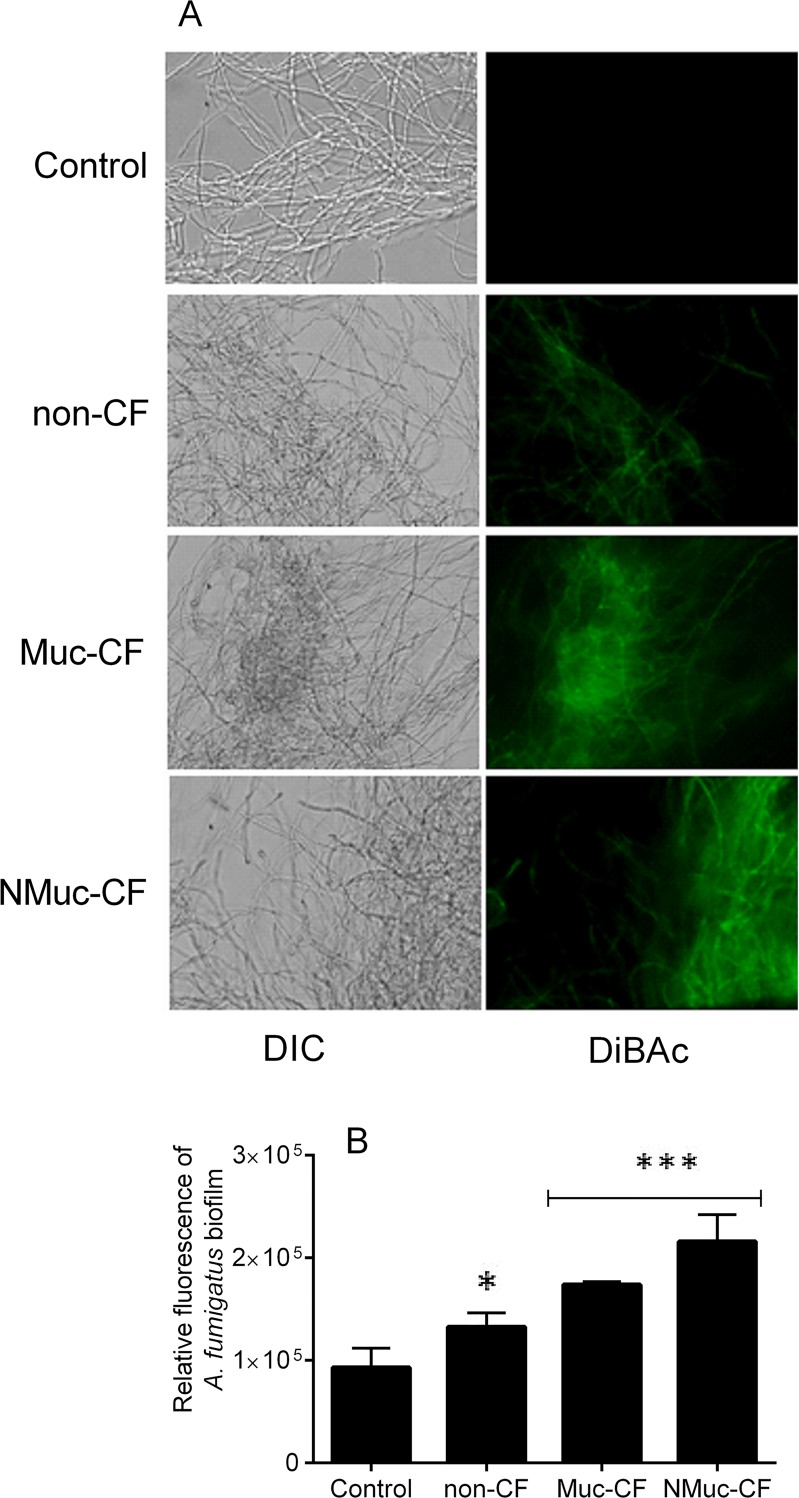
Membrane damaging action of *P*. *aeruginosa* biofilm culture filtrates (non-CF, Muc-CF and NMuc-CF) on preformed *A*. *fumigatus* biofilms, as shown by the morbidity stain DiBAC. (A) Fluorescence images of *A*. *fumigatus* preformed biofilms stained with DiBAC. (B) Relative fluorescence of preformed *A*. *fumigatus* biofilms stained with DiBAC. Bars represent fungicidal action as measured by DiBAC staining using spectrophotometry at 490 nm. Assays were performed in triplicate and results are presented as the mean of three replicates. Error bars represent the standard deviation of the mean. DIC: differential interference contrast. *p <0.05, ***p <0.0001 (compared with untreated controls).

Furthermore, we observed that DiBAC uptake, a marker of membrane damage, was higher in *A*. *fumigatus* preformed biofilms treated with biofilm culture filtrates from NMuc-CF or Muc-CF *P*. *aeruginosa* strains than in *A fumigatus* biofilms biofilms exposed to non-CF *P*. *aeruginosa* biofilm culture filtrates or in untreated control *A*. *fumigatus* biofilms. Specifically, this effect was more prominent in preformed *A*. *fumigatus* biofilms treated with NMuc-CF culture filtrates (2.4-fold higher DiBAC uptake) than in biofilms treated with Muc-CF culture filtrates (Fig 2A and 2B, p <0.001) or non-CF filtrates (1.4- to 1.8-fold higher) (p<0.0001) (Fig 2B). Non-CF P. aeruginosa filtrates were significantly different than controls

### Non-mucoid *P*. *aeruginosa* culture filtrates induce more pronounced apoptosis in preformed *A*. *fumigatus* biofilm

**Fig 3:**
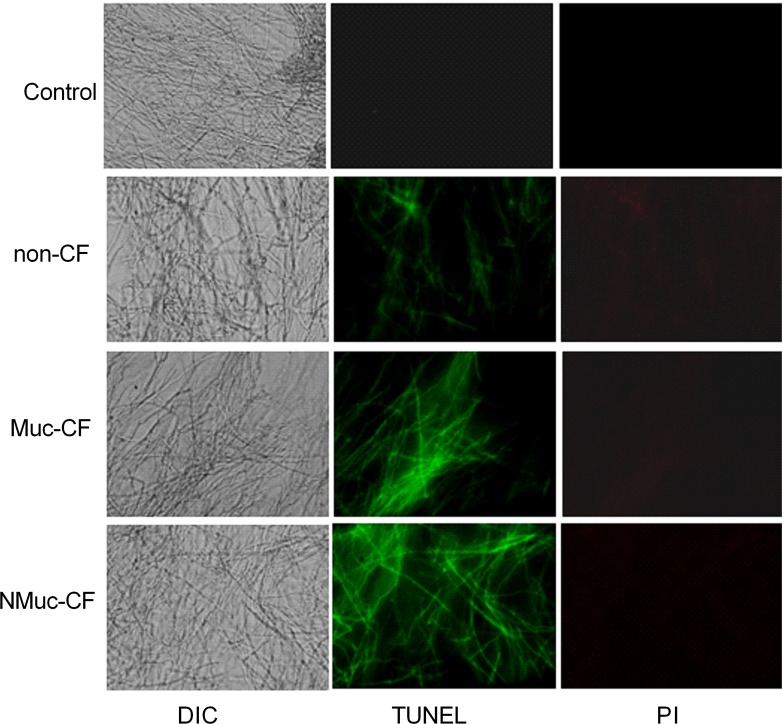
Culture filtrates of *P*. *aeruginosa* leads to DNA fragmentation and membrane disruption in preformed *A*. *fumigatus* biofilms, as shown by TUNEL assay and membrane integrity by propidium staining (PI). Fluorescence images of DNA fragmentation of preformed *A*. *fumigatus* biofilms treated with biofilm culture filtrates of non-CF, Muc-CF, and NMuc-CF *P*. *aeruginosa*. DIC: differential interference contrast.

To assess whether cell death in *A*. *fumigatus* biofilms following exposure to culture filtrate of *P*. *aeruginosa* biofilms (non-CF, Muc-CF, and NMuc-CF) occurs through induction of apoptosis, we performed TUNEL assays to detect DNA fragmentation. Compared with untreated control, exposure to non-CF, Muc-CF, or NMuc-CF *P*. *aeruginosa* culture filtrates resulted in increasing degrees of DNA fragmentation in preformed *A*. *fumigatus* biofilms (Fig 3).

### *P*. *aeruginosa* biofilm culture filtrate increase intracellular ROS accumulation in preformed *A*. *fumigatus* biofilms

**Fig 4:**
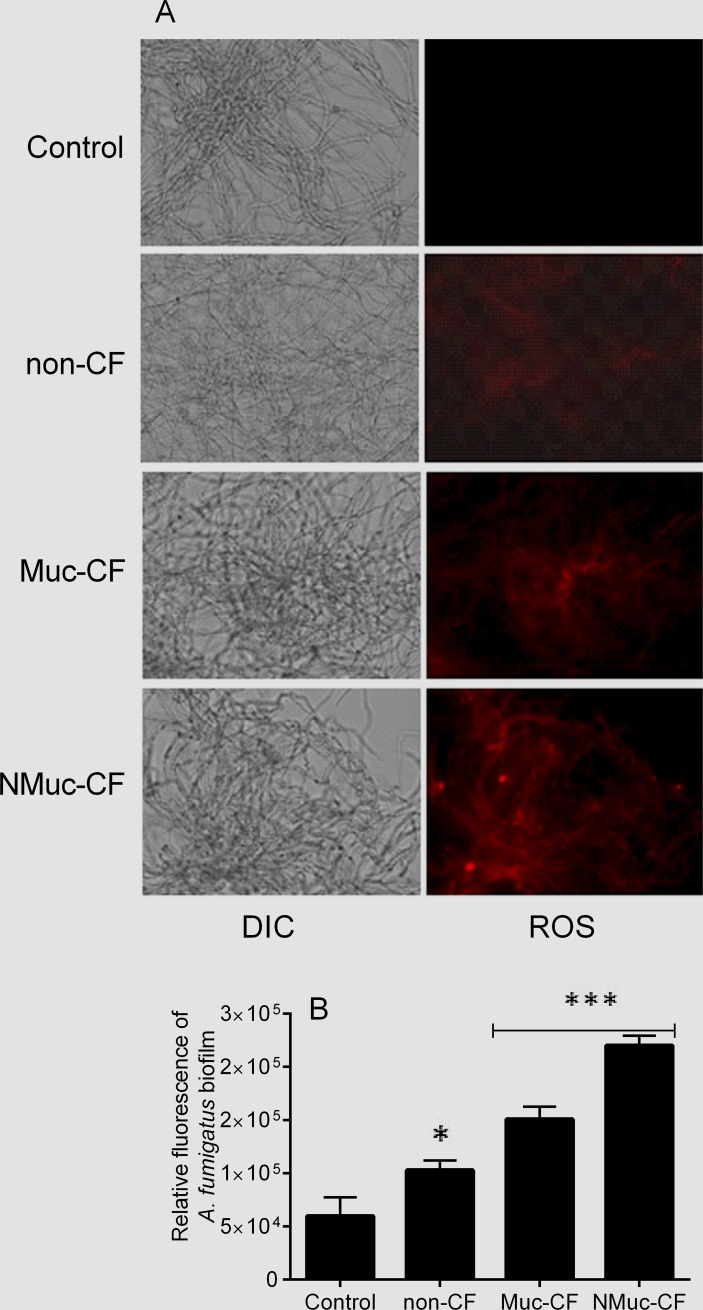
Intracellular reactive oxygen species (ROS) accumulation detected in preformed *A*. *fumigatus* biofilms treated with culture filtrates of *P*. *aeruginosa* biofilm, as shown by staining with dihydrorhodamine (DHR)-123. (A) Fluorescence images of preformed *A*. *fumigatus* treated with biofilm culture filtrates of non-CF, Muc-CF, and NMuc-CF *P*. *aeruginosa* stained with (DHR)-123. (B) Relative fluorescence of preformed *A*. *fumigatus* biofilms treated with *P*. *aeruginosa* biofilm culture filtrates stained with DHR 123. Bars represent ROS accumulation as shown by DHR 123 staining and by spectrophotometry at 490 nm. Assays were performed in triplicate and results are presented as the mean of three replicates. Error bars represent the standard deviation of the mean. DIC: differential interference contrast. *p<0.05, ***p<0.0001 (compared with untreated controls).

ROS play an important role as early initiators of apoptosis in yeasts and other filamentous fungi [11–16]. Using a fluorometric assay with DHR-123 staining, we found significantly higher intracellular ROS levels in preformed *A*. *fumigatus* biofilms treated with *P*. *aeruginosa* culture filtrate compared with untreated controls. Specifically, in preformed *A*. *fumigatus* biofilms treated with NMuc-CF, the relative fluorescence was 3.7-fold (p<0.0001) compared with Muc-CF (2.5-fold) and non-CF (1.7-fold) treated and untreated controls (Fig 4A and 4B). Muc-CF-treated biofilms showed significantly higher relative fluorescence than did non-CF treated biofilms (p <0.0001), as did non-CF treated biofilms compared to untreated controls (p <0.0001).

### *P*. *aeruginosa* biofilm culture filtrate decreases mitochondrial membrane potential in preformed *A*. *fumigatus* biofilms

**Fig 5:**
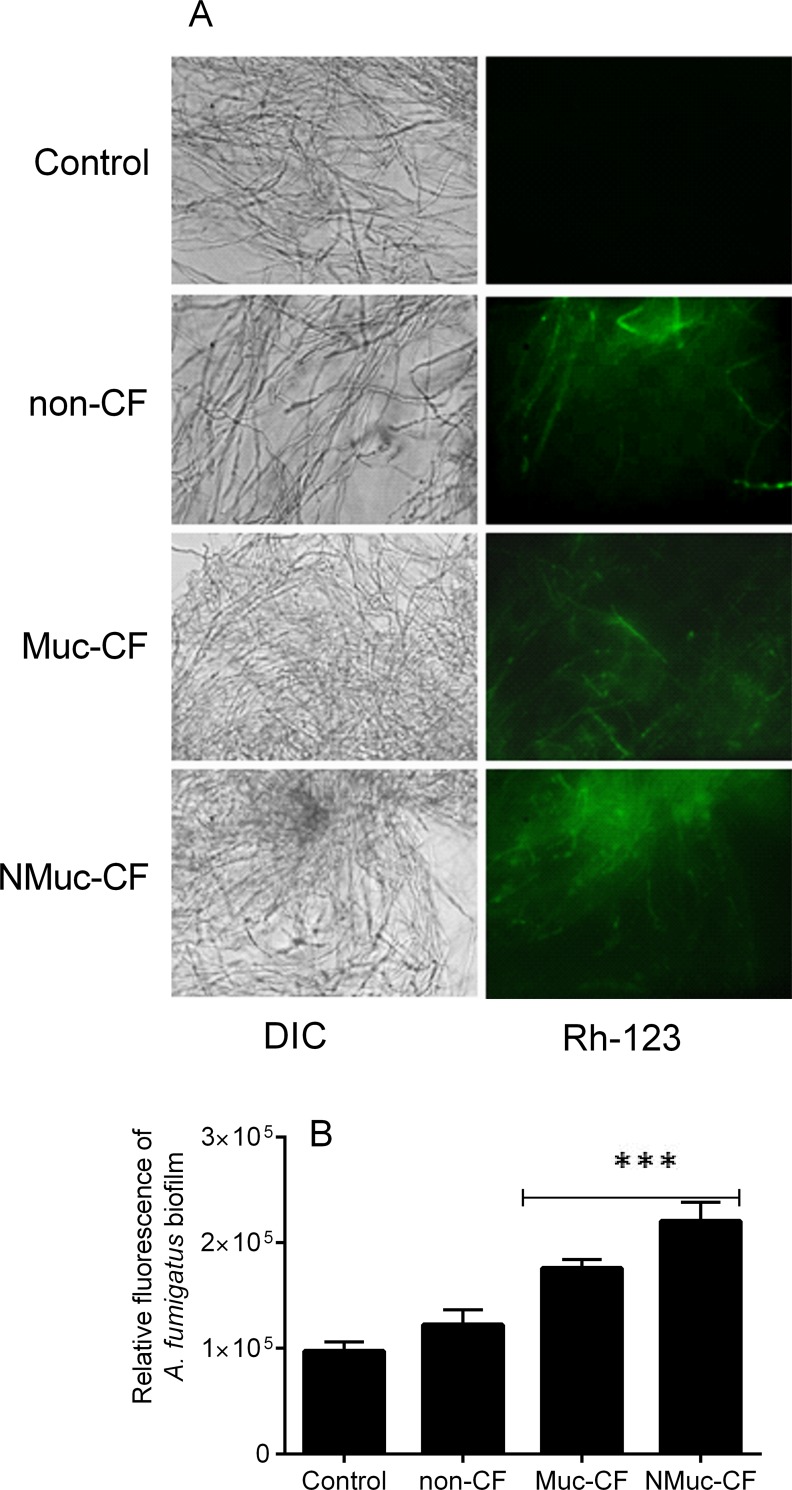
Depolarization of mitochondrial membrane potential as shown by rhodamine 123 staining in *A*. *fumigatus* preformed biofilms treated with *P*. *aeruginosa* biofilm culture filtrates. (A) Fluorescence images of preformed *A*. *fumigatus* biofilms treated with biofilm culture filtrates of non-CF, Muc-CF, and NMuc-CF *P*. *aeruginosa* stained with rhodamine 123. (B) Relative fluorescence of preformed *A*. *fumigatus* biofilms treated with *P*. *aeruginosa* biofilm culture filtrates stained with rhodamine 123. Bars represent mitochondrial membrane depolarization as shown by rhodamine 123 staining and by spectrophotometry at 490 nm. Assays were performed in triplicate and results are presented as the mean of three replicates. Error bars represent the standard deviation of the mean. ***p<0.0001 (compared with untreated controls).

Mitochondrial membrane depolarization was assessed by staining with rhodamine (Rh)-123, a fluorescent dye that is distributed in the mitochondrial matrix, as previously described [11–16]. *A*. *fumigatus* preformed biofilm treated with NMuc-CF or Muc-CF *P*. *aeruginosa* culture filtrates showed sharp decreases in mitochondrial potential (1.8 to 2.4-fold increase in fluorescence compared with untreated controls; p<0.0001), whereas the mitochondrial potential in the non-CF-treated *A*. *fumigatus* biofilm increased fluorescence by 1.25-fold c compared to untreated control (p<0.0001) (Fig 5A and 5B). The difference observed in the NMuc-CF-treated biofilm compared with control was significantly greater than that seen in the Muc-CF-treated biofilm (p <0.0001).

### Biofilm culture filtrates of *P*. *aeruginosa* activate caspase-like activity in *A*. *fumigatus* preformed biofilms

**Fig 6:**
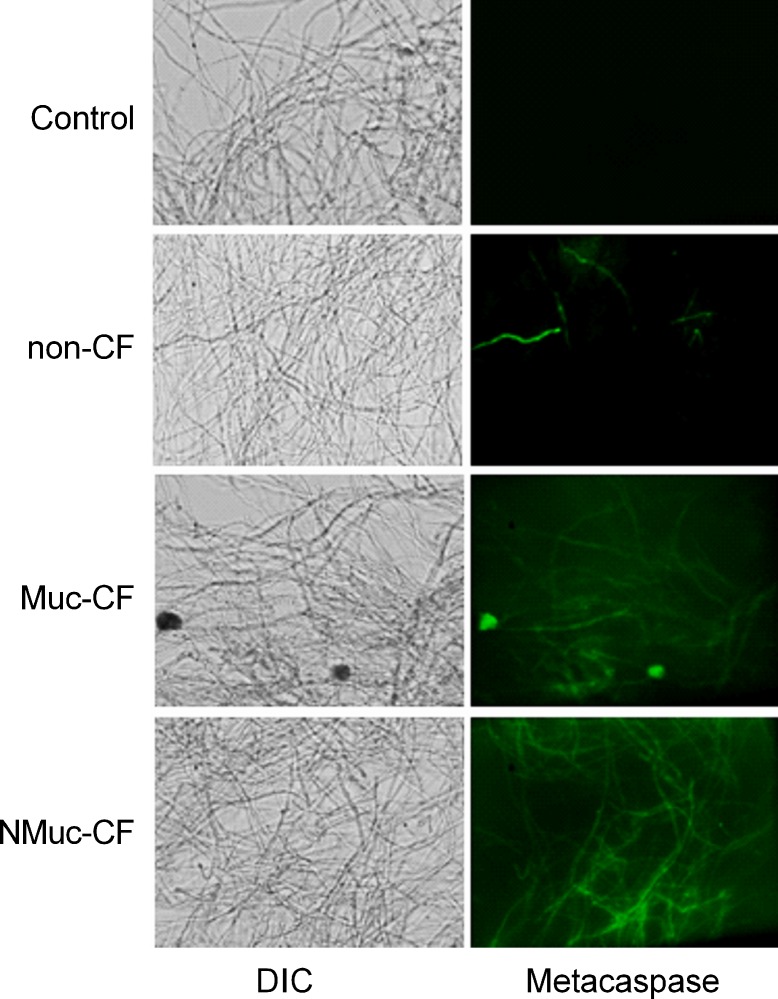
*A*. *fumigatus* preformed biofilms treated with *P*. *aeruginosa* biofilm culture filtrates activates caspase-like (metacaspase) activity. Fluorescent images depicting metacaspase activation of preformed *A*. *fumigatus* biofilm treated with culture filtrates of non-CF, Muc-CF, and NMuc-CF *P*. *aeruginosa*, as detected by staining with CaspACE FITC-VAD-FMK. DIC: differential interference contrast.

Metacaspases, orthologs of mammalian caspases that are found in fungi and plants, are activated in the early stages of apoptosis and play a central role in the apoptotic cascade [18–20]. We detected metacaspase activation using CaspACE FITC-VAD-FMK In Situ Marker. The cells of the preformed *A*. *fumigatus* biofilm treated with biofilm culture filtrates of *P*. *aeruginosa* showed green fluorescence, indicating activation of the marker and suggesting that exposure to *P*. *aeruginosa* biofilms may induce apoptosis in *A*. *fumigatus* by activating metacaspases (Fig 6). Again, the inhibitory effect of biofilm filtrates from NMuc-CF and Muc-CF Pa was greater compared to that of non-CF Pa filtrates.

## Discussion

Our study confirms that *P*. *aeruginosa* biofilm culture filtrates significantly compromise *A*. *fumigatus* preformed biofilm and that this effect is associated with apoptosis induction in preformed biofilms. Although studying more strains of each phenotype would have augmented the correlation between apoptosis and the different groups studied. In a previous study, each of the 3 phenotypic groups formed into tight cohorts with respect to effect on *A*. *fumigatus* metabolism [7], thus making it rational to pick representative isolates for extensive studies, as detailed here.

Apoptosis is, in general, initiated by multiple extracellular and intracellular stimuli. In filamentous fungi, apoptosis is associated with mitochondria depolarization, ROS production, and metacaspase activity [11–16, 21]. Many microbes produce virulence factors that induce apoptosis in host cells [22], and several antimicrobial agents have been observed to be proapoptotic agents in a number of filamentous fungi [22]. However, the nature of the signaling and effector pathways that regulate fungal apoptosis still remains unclear [21].

Previous investigations have suggested that secondary metabolites of *P*. *aeruginosa* are responsible for its inhibitory effect on *A*. *fumigatus* biofilms [5]. Secondary metabolites from *Pseudomonas*, such as phenazines, join a growing list of compounds (e.g., H_2_O_2_, amphotericin B, the antifungal protein PAF, sphingoid bases, itraconazole, and posaconazole) that are known to trigger an apoptotic response in fungi [11–16, 23, 24–27]. Briard et al. [28] showed that shift from vegetative growth to conidiation in *A*. *fumigatus* is associated with phenazine radicals and ROS accumulation during phenazine redox cycling, which showed a dual role of phenazine as toxic at higher levels and as sporulation signal at moderate level. Phenazine 1-carboxamide, a *Pseudomonas* metabolite, has been reported to induce apoptosis in the non-pathogenic Zygomycete *Benjaminiella poitrasii* [23], and phenazine-like compounds have been shown to eliminate other fungal competitors in mammalian host environments [23, 29]. *P*. *aeruginosa* has been shown to produce phenazines, present in CF sputum at concentrations between 1 and 100 μM, which are important in regulating *Aspergillus* development [30, 31]. Zheng et al. [31] have demonstrated that bacterial metabolites can act as sporulation signals affecting fungal development in mixed-species communities and that bacterial phenazine production modulates *P*. *aeruginosa*-*A*. *fumigatus* co-culture biofilm formation. Denial of iron appears to be another important mechanism of inhibition of *Aspergillus* by *Pseudomonas* [8].

Intracellular accumulation of ROS is another major stimulus for the induction of apoptosis in eukaryotes; in fact, it is considered one of the important hallmarks of apoptosis [24, 25]. ROS accumulation is understood to be a converging pathway underlying cellular damage induced by different types of stress, including oxidative stress [13, 21]. Antifungal agents primarily act through stimulating ROS accumulation in yeast and filamentous fungi [24, 32, 33, 34]. Quorum-sensing molecules are also thought to play an important role in determining pathogens’ ability to compete with each other for space and nutrients, and may contribute to persistent infections in the airways of CF patients [4]. The quorum-sensing molecule farnesol has been reported to induce apoptosis in fungi by promoting intracellular ROS generation [17, 35–38]. For example, Semighini et al. [17] reported that farnesol produced by *Candida albicans*, when co-cultured with *Aspergillus*, caused nuclear condensation and cell death of *Aspergillus*. Also, 3-oxo-C12 homoserine lactone, a quorum-sensing molecule from *P*. *aeruginosa*, which has structural similarity to farnesol, was shown to mimic the action of farnesol in *C*. *albicans* [38].

Instead of caspases, fungi have related proteases called metacaspases [20]. Apoptotic pathways in fungi can be either metacaspase-dependent or metacaspase-independent [39]. In our study, we show that *P*. *aeruginosa* culture filtrates inhibited growth of *A*. *fumigatus* preformed biofilm and triggered apoptosis via the induction of metacaspase activity, although the specific role of metacaspases needs to be investigated further.

Our results add to the growing evidence regarding the antagonistic interactions between fungal and bacterial biofilm communities. This study confirms that the inhibiting capacity of NMuc-CF *P*. *aeruginosa* filtrates was greater than that of Muc-CF and non-CF filtrates, and suggests correlation in the relative abilities of each to act by inducing apoptotic cell death. Dose correlation between the concentrations of specific metabolites and apoptosis has not been made, however concentration dependent dose responsiveness of *Pseudomonas* supernatants and metabolic inhibition of *A*. *fumigatus* has been previously detailed [7]. Future studies are needed to determine how *A*. *fumigatus* senses and responds to secondary metabolites from other microbial communities. Strategies targeting *Pseudomonas* with antibiotics in CF lungs need to be further explored in regards to the elimination of *Aspergillus* from respiratory secretions in these patients [40]. Research is also needed to discern the molecular mechanisms that govern apoptosis in *A*. *fumigatus* after *P*. *aeruginosa* culture filtrate treatment and to evaluate the potential of the metabolic products of *P*. *aeruginosa* as antifungal agents against *A*. *fumigatus*.
